# Supplementary material for: The interactome of intact mitochondria by cross-linking mass spectrometry provides evidence for coexisting respiratory supercomplexes
Source: Mol Cell Proteomics. 2017 Dec 8;17(2):216–32. doi: 10.1074/mcp.RA117.000470 (PMC5795388; doi:10.1074/mcp.RA117.000470)
Supplement: Supplemental Data [file supp_RA117.000470_133922_0_supp_23978_fzffwf.zip › spectra_annotation/mito_DR_spectra_annotation/276-1-11-1-5-1.pdf]

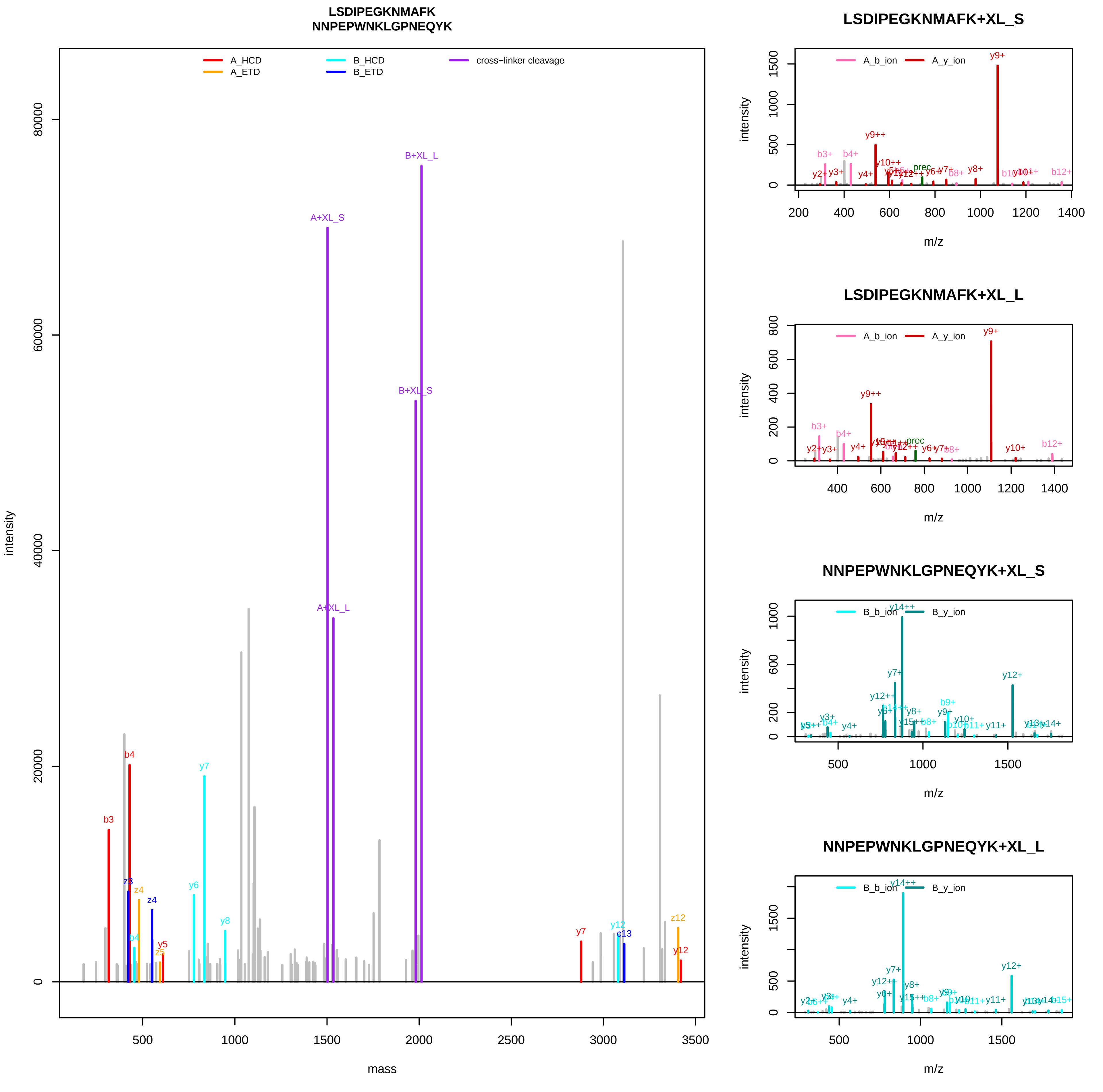
LSDIPEGKNMAFK
NNPEPWNKLGPNEQYK
A_HCD
A_ETD
B_HCD
B_ETD
cross−linker cleavage
intensity
mass
B+XL_L
A+XL_S
B+XL_S
A+XL_L
b3
b4
z3
z4
y5
z5
y6
y7
y8
y7
y12
c13
z12
y12
LSDIPEGKNMAFK+XL_S
LSDIPEGKNMAFK+XL_L
NNPEPWNKLGPNEQYK+XL_S
NNPEPWNKLGPNEQYK+XL_L
A_b_ion
A_y_ion
B_b_ion
B_y_ion
m/z
prec
y9+
y9++
y12+
y14++
